# Mortality of older acutely admitted medical patients after early discharge from emergency departments: a nationwide cohort study

**DOI:** 10.1186/s12877-021-02355-y

**Published:** 2021-07-02

**Authors:** Martin Aasbrenn, Christian Fynbo Christiansen, Buket Öztürk Esen, Charlotte Suetta, Finn Erland Nielsen

**Affiliations:** 1 CopenAge – Copenhagen Center for Clinical Age Research, University of Copenhagen, Copenhagen, Denmark; 2 Geriatric Research Unit, Department of Geriatric and Palliative Medicine, Copenhagen University Hospital, Bispebjerg and Frederiksberg, Copenhagen, Denmark; 3 Department of Clinical Epidemiology, Aarhus University Hospital, Aarhus, Denmark; 4 Geriatric Research Unit, Department of Medicine, Herlev-Gentofte Hospitals, Herlev, Denmark; 5 Department of Emergency Medicine, Copenhagen University Hospital, Bispebjerg and Frederiksberg, Copenhagen, Denmark; 6 Department of Emergency Medicine, Slagelse Hospital, Slagelse, Denmark; 7 Copenhagen Center for Translational Research, Copenhagen University Hospital, Bispebjerg and Frederiksberg, Copenhagen, Denmark

**Keywords:** Acute medicine, Anemia, Comorbidity, Constipation, Dehydration, Early discharge, Geriatrics, Heart failure, Length of stay, Respiratory failure

## Abstract

**Background:**

The mortality of older patients after early discharge from hospitals is sparsely described. Information on factors associated with mortality can help identify high-risk patients who may benefit from preventive interventions. The aim of this study was to examine whether demographic factors, comorbidity and admission diagnoses are predictors of 30-day mortality among acutely admitted older patients discharged within 24 h after admission.

**Methods:**

All medical patients aged ≥65 years admitted acutely to Danish hospitals between 1 January 2013 and 30 June 2014 surviving a hospital stay of ≤24 h were included. Demographic factors, comorbidity, discharge diagnoses and mortality within 30 days were described using data from the Danish National Patient Registry and the Civil Registration System. Cox regression was used to estimate adjusted hazard ratios (aHR) with 95% confidence intervals (CI) for all-cause mortality.

**Results:**

A total of 93,295 patients (49.4% men) with a median age of 75 years (interquartile range: 69–82 years), were included. Out of these, 2775 patients (3.0%; 95% CI 2.9–3.1%) died within 30 days after discharge. The 30-day mortality was increased in patients with age 76–85 years (aHR 1.59; 1.45–1.75) and 86+ years (aHR 3.35; 3.04–3.70), male gender (aHR 1.22; 1.11–1.33), a Charlson Comorbidity Index of 1–2 (aHR 2.15; 1.92–2.40) and 3+ (aHR 4.07; 3.65–4.54), and unmarried status (aHR 1.17; 1.08–1.27). Discharge diagnoses associated with 30-day mortality were heart failure (aHR 1.52; 1.17–1.95), respiratory failure (aHR 3.18; 2.46–4.11), dehydration (aHR 2.87; 2.51–3.29), constipation (aHR 1.31; 1.02–1.67), anemia (aHR 1.45; 1.27–1.66), pneumonia (aHR 2.24; 1.94–2.59), urinary tract infection (aHR 1.33; 1.14–1.55), dyspnea (aHR 1.57; 1.32–1.87) and suspicion of malignancy (aHR 2.06; 1.64–2.59).

**Conclusions:**

Three percent had died within 30 days. High age, male gender, the comorbidity burden, unmarried status and several primary discharge diagnoses were identified as independent prognostic factors of 30-day all-cause mortality.

## Background

The number of adults aged above 65 years is increasing, and a substantial proportion will need care and treatment for acute medical diseases each year [1]. Moreover, hospitalizations are associated with high risks of adverse events and readmissions [2]. Thus, concerns that the burden on health care systems might reach unmanageable proportions are regularly raised [3]. Acute medical disorders that need specialized evaluation can either be handled as outpatient contacts, or as longer hospital admissions. A third option is to hospitalize the patients to secure the diagnosis and the treatment plan in short term units, and then discharge them early to their own homes or to rehabilitation units [4].

Short hospital stays may increase the bed turnover and release capacity in the departments. Additionally, increased length of stay (LOS) has been shown to be associated with increased risk of hospital-acquired complications such as infections [5, 6]. Therefore, LOS is often used to assess the efficiency of hospital treatments. Old people are vulnerable having a burden of comorbidities and multiple medications, and do often need help doing everyday activities because of a reduced functional capacity [1, 7]. We have previously published a 30-day mortality rate of 3 % among elderly acutely admitted medical patients who were discharged within 24 h [8]. However, more research on the safety of early discharge and predictors of adverse events after discharge of older patients is needed [9]. Clinicians should pay attention to patients with characteristics associated with increased risk of mortality during the planning of early discharge of acutely admitted old patients. Further on, if modifiable, these risk factors should also be considered and included in studies of the effect of specific interventions on the risk factors aiming to reduce mortality after early discharge. The aim of our study was therefore to examine if demographic factors, comorbidities and admission diagnoses are associated with 30-day mortality among acutely admitted older patients discharged within 24 h after admission.

## Methods

### Design and setting

The methods used to identify patients and to collect data have previously been described in a study of readmission of older patients after early discharge [8].

The Danish health care system provides tax-funded health care to all Danish residents [10]. The national emergency health care service is carried out by 21 acute care hospitals staffed with specialists on a 24-h basis [10], whereas privately funded hospitals in Denmark do not have acute patient intake [10]. General practitioners refer most of the acutely admitted patients.

### Data sources

This cohort study was conducted using the Danish National Patient Registry (DNPR) [11], covering all Danish hospitals since 1977, and the Danish Civil Registration System (CRS) [12], covering individual-level information on all Danish residents since 1968. The different national administrative registers can be linked using the unique personal identification number assigned to all Danish residents. The DNPR provides nationwide registration of detailed administrative and clinical data [11]. The registry contains information on all hospital contacts in Denmark, surgical procedures, major treatments performed, one primary discharge diagnosis, and secondary inpatient and outpatient discharge diagnoses. The diagnoses in the registry are coded by use of the Danish language version of the International Classification of Diseases 10th edition (ICD-10). The CRS contains up-to-date information on vital status, marital status and emigration [12].

### Study population

We used the DNPR to identify all adult medical patients (≥ 18 years) admitted acutely to Danish hospitals between 1 January 2013 and 30 June 2014 and discharged within 24 h after admission.

Patients were excluded from the study if they had acute contacts to an emergency room without admission to an emergency department or medical ward, if they died within the first 24 h during their stay, or were transferred to other hospitals.

### Covariates

Using the DNPR, we have characterized all patients by age and gender. For the index admission we obtained data from the DNPR on the admission and discharge time, Charlson Comorbidity Index (CCI) [13], primary discharge diagnoses reflecting the main reason for hospitalization, secondary diagnoses, malignancy, R-diagnoses (used if symptoms, signs and abnormal findings are not classified elsewhere) and Z-diagnoses (used when a diagnosis has not yet been confirmed). Information on civil status at the index admission time and vital status during the study period were obtained from the CRS.

The comorbidity burden was assessed by use of CCI, which was computed based on all primary and secondary discharge diagnoses that had been registered in the DNRP since 1977 [14]. The CCI score was divided into three levels: low (score 0), moderate (score 1–2), and high (score 3+).

### Statistical analysis

The outcome was all-cause mortality (time to death) within the first 30 days of discharge after the index admission.

Initially the population was described (age, gender, CCI, marital status, discharge diagnoses) according to survival status, and time to death was analyzed according to the different baseline characteristics. Next, univariate and multivariate analyzes of the rate of death were performed.

Categorical variables are reported as counts and percentages. Continuous data are presented as medians with interquartile ranges (IQR). Differences between proportions or medians are described using exact differences between proportions with 95% confidence intervals (CI) and 95% confidence intervals of the median differences, respectively.

We followed patients from the date of discharge from the index admission, until the end of the follow-up period, emigration or death, whichever came first. We used Cox regression to estimate adjusted hazard ratios (aHRs) with 95% confidence intervals (CI) for covariates in multivariable models. Initially, HRs for mortality were estimated in a regression model including age, gender, marital status, and CCI. Subsequently, we computed the HRs for mortality including main reason for hospitalization (primary diagnosis) as covariate in the model with further adjustment for age, gender, marital status and CCI. The assumption of proportional hazards for all Cox regression models was assessed graphically using log (−log (survival probability))-plots and found appropriate.

Analyses were performed using the statistical software package Stata, version 15.1 (StataCorp, College Station, TX, USA).

## Results

### Population

A total of 1,096,637 patients were acutely admitted during the study period. A total of 551,185 (50.3%) were medical patients, and 243,439 (44.2%) were discharged within 24 h after admission.

This study included all medical patients aged ≥65 years (*n* = 93,295) discharged within 24 h. The median age was 75 years (IQR 69–82), 49.4% were male and 50.3% were married. A total of 22,206 (23.8%) of the patients had a high comorbidity burden (CCI 3+) (Table 1). A total of 18,213 (19.5%; 95% CI 19.2–19.7%) had a history of cancer before the index admission.

**Table 1:** 30-day mortality and time to death according to baseline characteristics

	Mortality N (%, 95 CI)	Time to death Median days (IQR)
Median age, years (IQR)	81 (73–87)	
Age groups		
65–75 years (*n* = 44,466)	780 (1.8; 1.6–1.9)	15 (7–22)
76–85 years (*n* = 32,760)	997 (3.0; 2.9–3.2)	14 (8–22)
86+ years (*n* = 16,069)	998 (6.2, 5.8–6.6)	12.5 (6–21)
Gender		
Male (*n* = 46,188)	1467 (3.2; 3.0–3.3)	13 (6–22)
Female (*n* = 47,107)	1308 (2.8; 2.6–2.9)	15 (7–22)
CCI		
0 (*n* = 34,670)	445 (1.3; 1.2–1.4)	12 (6–21)
1–2 (*n* = 36,419)	1088 (3.0; 2.8–3.2)	13 (6–21)
3+ (*n* = 22,206)	1.242 (5.6; 5.3–5.9)	15 (7–23)
Marital Status		
Married (n = 46,965)	1139 (2.4; 2.3–2.6)	15 (7–22)
Not Married (n = 46,330)	1636 (3.5; 3.4–3.7)	13 (6–22)

*CCI* Charlson Comorbidity Index, *CI* confidence interval, *IQR* interquartile range

### Mortality

Among all acutely admitted patients discharged within 24 h, 2775 (3.0%; 95% CI 2.9–3.1%) died within the first 30 days after the admission. The median time to death was 14 days (IQR 7–22).

### Baseline characteristics according to survival status

The baseline characteristics according to 30-day survival are given in Tables 1 and 2. The patients who died were older (81 years vs. 75 years; 95% CI for the difference in medians: 4–5 years). The mortality was higher in males compared to females (3.2% vs. 2.8%; 95% CI for the difference: 0.2–0.6%), in unmarried compared to married (3.5% vs. 2.4%; 95% CI for the difference: 0.9–1.3%) and in patients with moderate to high comorbidity burden (CCI > 0) compared to patients with CCI 0 (4.0% vs. 1.3, 95% CI for the difference: 2.5–2.9%). The median time to death was shorter among patients aged 86+ (12.5 days) compared to patients aged 65–85 years (14 days) (median difference 1 day, 95% CI 1–2) (Table 1). Median time to death did not differ significantly between females and males (15 vs. 13 days, 95% CI for the difference in medians: 0–2 days), between patients with low (12 days) and moderate to high comorbidity burden (14 days) (median difference 1 day, 95% CI 0–2) and between married (15 days) and unmarried patients (13 days) (median difference 1 day; 95% CI 0–2).

**Table 2:** 30-day mortality and time to death according to primary discharge diagnoses

Discharge diagnoses	Mortality N (%; 95% CI)	Time to death, median days (IQR)
Diseases of the cardiovascular system		
Angina pectoris (*n* = 1901)	11 (0.6; 0.3–1.0)	16 (11–22)
Atrial fibrillation (*n* = 7187)	59 (0.8; 0.6–1.1)	15 (8–22)
Deep vein thrombosis (*n* = 1324)	18 (1.4; 0.8–2.1)	14.5 (9–26)
Hypertension (*n* = 1622)	7 (0.4; 0.2–0.9)	9 (7–22)
Heart failure (1004)	61 (6.1; 4.7–7.7)	10 (6–18)
Diseases of the respiratory system		
COPD (*n* = 3088)	111 (3.6; 3.0–4.3)	14 (6–21)
Respiratory failure (*n* = 564)	60 (10.6; 8.2–13.5)	7 (1–14)
Diseases of the nervous system		
TIA (*n* = 1441)	13 (0.9; 0.5–1.5)	21 (17–25)
Epilepsy (*n* = 650)	5 (0.8; 0.3–1.8)	13 (12–16)
Endocrine and nutritional diseases		
Diabetes (*n* = 598)	23 (3.9; 2.5–5.7)	18 (8–25)
Dehydration (*n* = 2167)	231 (10.7; 9.4–12.0)	12 (6–19)
Diseases of the digestive system		
Dyspepsia (*n* = 358)	NA	NA
Esophagitis (*n* = 53)	NA	NA
Constipation (1502)	66 (4.4; 3.4–5.6)	18 (9–24)
Ulcer without bleeding (*n* = 49)	NA	NA
Hematological diseases		
Anemia (*n* = 4168)	240 (5.8; 5.1–6.5)	18 (11–25)
Infectious disease		
Erysipelas (*n* = 769)	14 (1.8; 1.0–3.0)	14.5 (3–25)
Gastroenteritis (*n* = 652)	10 (1.5; 0.7–2.8)	7 (6–9)
Pneumonia (*n* = 2711)	197 (7.3; 6.3–8.3)	11 (5–19)
Urinary Tract Infection (*n* = 3468)	174 (5.0; 4.3–5.8)	14 (7–22)
Spinal disease/arthritis		
Back Pain (*n* = 1028)	19 (1.8; 1.1–2.9)	14 (6–25)
Arthritis (*n* = 166)	NA	NA
Mental and behavioral disorders		
Misuse of drugs or alcohol (*n* = 1078)	26 (2.4; 1.6–3.5)	14.5 (7–22)
R-diagnoses^a^		
Chest pain (*n* = 4157)	36 (0.9; 0.6–1.2)	14.5 (822.5)
Dyspnea (*n* = 2450)	133 (5.4; 4.6–6.4)	13 (7–21)
Fever (*n* = 688)	20 (2.9; 1.8–4.5)	10.5 (4–19.5)
Headache (*n* = 569)	3 (0.5; 0.1–1.5)	15 (11–17)
Pains in neuromuscular system (*n* = 1035)	25 (2.4; 1.6–3.6)	14 (5–26)
Abdominal pain (*n* = 4916)	116 (2.4; 2.0–2.8)	16 (9–23)
Other pain (*n* = 1246)	48 (3.9; 2.9–5.1)	12.5 (5.5–21.5)
Vertigo (*n* = 1984)	18 (0.9; 0.5–1.4)	15.5 (8–23)
Other R-diagnoses (n = 10,822)	440 (4.1; 3.7–4.5)	15 (7–23)
Z-diagnoses^b^		
Malignancy (*n* = 1526)	75 (4.9; 3.9–6.1)	21 (10–26)
Central-nervous diseases (*n* = 1841)	44 (2.4; 1.7–3.2)	14 (6–22)
Heart diseases (*n* = 3297)	30 (0.9; 0.6–1.3)	19.5 (10–25)
Myocardial infarction (*n* = 5705)	43 (0.8; 0.6–1.0)	17 (8–23)
Rehabilitation (8035)	227 (2.8; 2.5–3.2)	11 (1–29)
Other Z-diagnoses (*n* = 3986)	123 (3.1; 2.6–3.7)	11 (6–20)

*CI* confidence interval, *COPD* chronic obstructive pulmonary disease, *IQR* interquartile range, *NA* not available (data not presented when there are less than 5 events in the group due to privacy concerns), *TIA* transient ischemic attack

^a^Used if symptoms, signs and abnormal clinical and laboratory findings are not elsewhere classified. ^b^Used when a diagnosis has not yet been confirmed

### Discharge diagnoses according to survival status

Mortality rates according to discharge diagnoses are presented in Table 2. The mortality was relatively high among patients with heart failure (6.1%), respiratory failure (10.6%), dehydration (10.7%), constipation (4.4%), anemia (5.8%), pneumonia (7.3%), urinary tract infection (5.0%) dyspnea (5.4%) and suspicion of malignancy (4.9%) (Table 2). Compared to all other patient groups we found that the median time to death was shorter in patients with respiratory failure (median difference 5 days, 95% CI 3–7 days), pneumonia (median difference 2 days; 95% CI 1–4 days), and the Z-diagnosis rehabilitation (median difference 2 days; 95% CI 1–3 days). Median time to death was longer in patients with anemia (median difference 4 days; 95% CI 2–5 days) and with suspicion of malignancy (median difference 4 days; 95% CI 2–6 days) (Table 2).

### Prognostic factors for mortality

In a regression model only including age, gender, CCI and marital status as covariates, we found that the age groups 76–85 years (aHR 1.59; 95% CI 1.45–1.75) and 86+ years (aHR 3.35; 95% CI 3.04–3.70) (65–75 years as reference), male gender (aHR 1.22, 95% CI 1.13–1.33), unmarried status (aHR 1.17 (95% CI 1.08–1.27), CCI 1–2 (aHR 2.15; 95% CI 1.92–2.40) and CCI 3+ (aHR 4.07; 95% CI 3.65–4.54) (CCI 0 as reference) were independent prognostic factors for mortality (Table 3).

**Table 3:** Hazard ratios for 30-day mortality according to age, gender, comorbidity and marital status, unadjusted and adjusted for the three other variables in the model

Baseline characteristics	Unadjusted HR (95% CI)	Adjusted HR (95% CI)^a^
Age (years)		
65–75	Reference	Reference
76–85	1.75 (1.59–1.92)	1.59 (1.45–1.75)
86+	3.62 (3.30–3.98)	3.35 (3.04–3.70)
Gender		
Female	Reference	Reference
Male	1.15 (1.06–1.23)	1.22 (1.13–1.33)
Charlson Comorbidity Index		
0	Reference	Reference
1–2	2.35 (2.10–2.62)	2.15 (1.92–2.40)
3+	4.44 (4.00–4.95)	4.07 (3.65–4.54)
Marital Status		
Married	Reference	Reference
Not Married	1.47 (1.36–1.58)	1.17 (1.08–1.27)

*HR* hazard ratio, *CI* confidence interval.^a^Adjusted for other three variables in the model.

In a second regression model with discharge diagnoses as covariates and after adjustment for age, gender, CCI and marital status, we found that heart failure (aHR 1.52; 95% CI 1.17–1.95), respiratory failure (aHR 3.18; 95% CI 2.46–4.11), dehydration (aHR 2.87; 95% CI 2.51–3.29), constipation (aHR 1.31; 95% CI 1.02–1.67), anemia (aHR 1.45; 95% CI 1.27–1.66), pneumonia (aHR 2.24; 95% CI 1.94–2.59), urinary tract infection (aHR 1.33; 95% CI 1.14–1.55), dyspnea (aHR 1.57; 95% CI 1.32–1.87) and suspicion of malignancy (aHR 2.06; 95% CI 1.64–2.59) were independent prognostic factors for 30-day mortality. Covariates associated with a reduced risk of 30-day mortality were the R-diagnoses chest pain (aHR 0.31; 95% CI 0.22–0.42), headache (aHR 0.20; 95% CI 0.07–0.64) and vertigo (aHR 0.32; 95% CI 0.20–0.51) (Table 4).

**Table 4:** Hazard ratios for 30-day mortality with the discharge diagnosis as covariate, unadjusted and adjusted for age, gender, comorbidity and marital status

Discharge diagnoses^a^	Unadjusted HR (95% CI)	Adjusted HR^b^ (95% CI)
Diseases of the cardiovascular system		
Angina pectoris	0.19 (0.11–0.34)	0.20 (0.11–0.37)
Atrial fibrillation	0.26 (0.20–0.33)	0.38 (0.29–0.50)
Deep vein thrombosis	0.45 (0.29–0.72)	0.53 (0.34–0.85)
Hypertension	0.14 (0.07–0.30)	0.18 (0.09–0.38)
Heart failure	2.11 (1.64–2.72)	1.52 (1.17–1.95)
Diseases of the respiratory system		
COPD	1.22 (1.01–1.48)	1.08 (0.89–1.30)
Respiratory failure	3.85 (2.98–4.97)	3.18 (2.46–4.11)
Diseases of the nervous systemTIA	0.30 (0.17–0.51)	0.35 (0.20–0.60)
Epilepsy	0.26 (0.11–0.61)	0.29 (0.12–0.70)
Endocrine and nutritional diseases		
Diabetes	1.30 (0.86–1.95)	1.07 (0.71–1.61)
Dehydration	4.00 (3.50–4.57)	2.87 (2.51–3.29)
Diseases of the digestive system		
Dyspepsia	0.28 (0.09–0.86)	0.33 (0.11–1.01)
Esophagitis	–	
Constipation	1.50 (1.17–1.90)	1.31 (1.02–1.67)
Ulcer without bleeding	–	
Hematological diseases		
Anemia	2.04 (1.80–2.33)	1.45 (1.27–1.66)
Infectious disease		
Erysipelas	0.61 (0.36–1.03)	0.62 (0.36–1.04)
Gastroenteritis	0.51 (0.28–0.95)	0.55 (0.29–1.01)
Pneumonia	2.62 (2.27–3.03)	2.24 (1.94–2.59)
Urinary Tract Infection	1.75 (1.50–2.04)	1.33 (1.14–1.55)
Spinal disease/arthritis		
Back Pain	0.62 (0.39–0.97)	0.71 (0.45–1.11)
Arthritis	0.40 (0.10–1.60)	0.51 (0.13–2.05)
Mental and behavioral disorders Misuse of drugs or alcohol	0.81 (0.55–1.19)	1.01 (0.68–1.48)
R-diagnoses^c^		
Chest pain	0.28 (0.20–0.39)	0.31 (0.22–0.42)
Dyspnea	1.89 (1.59–2.25)	1.57 (1.32–1.87)
Fever	0.98 (0.63–1.52)	1.03 (0.66–1.60)
Headache	0.17 (0.06–0.54)	0.20 (0.07–0.64)
Pains in neuromuscular system	0.81 (0.55–1.20)	0.72 (0.49–1.08)
Abdominal pain	0.78 (0.65–0.94)	0.86 (0.72–1.04)
Other pain	1.30 (0.98–1.74)	1.26 (0.95–1.67)
Vertigo	0.30 (0.19–0.47)	0.32 (0.20–0.51)
Other R-diagnoses	1.44 (1.30–1.60)	1.32 (1.19–1.46)
Z-diagnoses^d^	1.67 (1.33–2.11)	2.06 (1.64–2.59)
Malignancy	0.80 (0.59–1.08)	0.84 (0.62–1.13)
Central-nervous diseases	0.30 (0.21–0.42)	0.36 (0.25–0.51)
Heart diseases	0.24 (0.18–0.32)	0.26 (0.20–0.36)
Myocardial infarction	0.95 (0.83–1.08)	0.96 (0.84–1.10)
RehabilitationOther Z-diagnoses	1.04 (0.87–1.25)	1.03 (0.86–1.24)

*COPD* chronic obstructive pulmonary disease, *CI* confidence interval, *HR* hazard ratio, *TIA* transient ischemic attack

^a^The reference is other (coded zero) in the individual diagnosis group (coded 1)

^b^Hazard ratio for the discharge diagnosis adjusted for age, gender, Charlson Comorbidity Index and marital status

^c^Used if symptoms, signs and abnormal clinical and laboratory findings are not elsewhere classified. ^d^Used when a diagnosis has not yet been confirmed

## Discussion

In this nationwide cohort including 93,295 acutely admitted, older medical patients discharged within 24 h, only 3% of the patients died within the 30 days following discharge. The 30-day mortality rate after discharge from hospital with a medical diagnosis and variable LOS has in other studies been around 5% [15, 16]. Very short hospital stays have previously been problematized as a risky approach [17, 18]. Our data with a relatively low all-cause mortality rate indicate that the practice and selection of older patients for early discharge in Denmark in 2013 and 2014 was reasonably safe.

Male gender had in our study a 22% increased risk of death compared to females. Male gender has been associated with many negative health outcomes in older age, including earlier onset of chronic diseases, more readmissions and shorter survival at nursing homes [1, 8, 19, 20]. Worldwide, men do have shorter lifespan and higher rates of lethal conditions as heart disease and stroke than women, partly due to behavioral differences [21]. However, the mortality gap between men and women has been reduced in the recent decades [22]. To what extent the difference in mortality between older men and women after early discharge is due to differences that could be changed, such as health seeking behavior, social factors or psychiatric conditions, needs further research.

As presumed from the existing literature [20] and in accordance with older people having higher rates of coronary heart disease, diabetes, stroke, cancer and respiratory diseases [1], our study showed significant mortality differences across the three age groups with highest mortality rate among the oldest patients. The mortality was also higher among unmarried individuals. Associations between unmarried or single-living status and adverse outcomes after hospital admissions have also been shown in other studies [23, 24]. Unfortunately, it is not possible from the present data to investigate the proportion of unmarried older patients who lived alone, which previously has been associated with increased mortality and morbidity not least among older people [25]. Complex causal mechanisms behind the association between single living and mortality have been suggested [26]. Clarification of whether patients live alone and identification of social factors that can be improved could be included in the discharge planning process of elderly unmarried patients.

The number of comorbid conditions was a prognostic factor in our study, as in earlier research with a positive gradient among the number of diseases and mortality [27]: We found a mortality rate of 1.3% in patients with CCI 0, increasing to over 5% in patients with CCI 3+. Previously, we have found an association between CCI and readmission [8]. These findings indicate a need to take chronic disorders into consideration during discharge planning, both concerning medical treatment, follow-up and care. Community nursing has been associated with lower mortality after discharge. Unfortunately, we did not have data about community nursing in our study [9].

The mortality rates for patients with heart failure and respiratory failure were 6.1 and 10.6%, respectively, and both conditions were independent prognostic factors for mortality. High 30-day mortality rates among patients with heart failure (around 10%) and patients with respiratory failure (around 20%) were also observed in a nationwide Danish study of all medical admissions (both with short and long LOS) [16]. Direct comparison of these mortality rates is problematic because patients discharged within 24 h after discharge is a selected group and not comparable to a group with longer LOS. Regardless of the LOS, early discharged patients with heart failure or respiratory failure have substantial high 30-day mortality rates. It is beyond the scope of this paper to describe treatment of heart failure patients. However, to ensure optimal treatment with documented effect on survival and readmission, professionals are recommended to follow the specific recommendations in the guidelines regarding treatment, monitoring and follow-up of the older patient with heart failure [28].

Patients with pneumonia and urinary tract infection had mortality rates of 7.3 and 5.0%, and were independent prognostic factors of death. In the light of the early discharge we assume that the infectious diseases were uncomplicated without signs of sepsis, which is supported by the relatively low mortality rates compared to patients with complicated infections such as sepsis [29]. However, the mortality rates are not negligible, and it is noticeable that patients with pneumonia had a shorter time to death. In a previous study [8] from our group it was shown that older patients with pneumonia and urinary tract infections had an increased risk of readmission in the 30-day period after discharge. We do not have data on the causes of readmission and death, however, the present results indicate a need for increased focus on patients with these infectious diseases during discharge planning, to optimize treatment and out of hospital care to reduce the risk of complications after discharge.

Non-specific diagnoses or R-diagnoses (symptoms and abnormal findings, not elsewhere classified) are frequently used as discharge diagnosis in patients acutely admitted to medical departments in Denmark [30]. Patients discharged with the R-diagnosis dyspnea in our study had a mortality rate of 5.4%, and dyspnea was independently associated with death. Dyspnea was also associated with increased risk of readmission [8]. We are unable to conclude on the causes of dyspnea based on our data. However, the increased risk of death and readmission among patients discharged with the non-specific dyspnea diagnosis may indicate underlying unrecognized diseases and need of more extensive somatic diagnostic work-up prior to discharge.

Dehydration and constipation are common health problems among older adults [31, 32] and were identified as independent prognostic factors of death in our study. High mortality rates after hospitalizations for dehydration has also been observed in other Danish cohorts [16]. Dehydration and constipation can usually be treated without hospitalization by use of simple home remedies and attention to nutrition, lifestyle and medications [32, 33]. In addition to underlying disease, dehydration and constipation may also indicate suboptimal care outside the hospital with increased risk of a new deterioration at home after discharge. These discharge diagnoses and their causes should therefore be taken into account during the discharge planning.

More than 5 % of the patients discharged with the diagnosis anemia died within 30 days after discharge, and anemia was an independent prognostic factor of death. Anemia is very common in geriatric patients and may be a proxy for serious chronic disease such as cancer or a specific treatable condition [34]. In our study, anemia remained important prognostic information after adjustment for CCI (Table 4). We cannot investigate causes of anemia and whether the disorder was due to chronic or acute illness in the current data set. Further exploration of the associations between different types anemia and mortality is warranted to identify potentially modifiable factors associated with death.

The organization of palliative care is very different between countries. In Denmark, there are two types of specialized palliative care units; hospital-based palliative care and hospices, both of which are free of charge [35]. There are 48 palliative beds per million inhabitants, which is lower than the recommendations from the European Association for Palliative Care [35]. It is likely that some of the deaths registered in our study occurred in patients who received palliative care. We have not taken terminal phase of diseases into account in our study.

The R-diagnoses chest pain, headache and vertigo were associated with a low risk of 30-day mortality. When patients present in primary care with chest pain or vertigo, it will frequently lead to a hospital referral to evaluate whether the symptom is due to an acute cardiovascular event. Such a vascular event can often be excluded within 24 h and with finding of more benign causes as explanation for the symptoms. This may explain why these R-diagnoses had significantly lower 30-day mortality rates than many of the other diagnoses.

### Strengths and limitations

The number of included patients was high, which implies a low risk of random errors. The study was based on a population-based database that contained all the acute medical hospital admissions in the country in a specific time period. This registration is complete as private hospitals in Denmark do not have acute patient intake. All individuals with permanent residence in Denmark are assigned a unique personal identification number which makes complete follow up in the CRS registry with vital status possible. However, we cannot exclude that insufficient capacity of home care outside the hospital, and inconvenient time (nights) of discharge, might have a negative impact on the discharge process in general and may have caused a delay of the time of discharge among some patients (e.g. frail older people) resulting in discharge times more than 24 h after admission for these patients. Consequently, selection bias may have been introduced into our study.

There are other limitations to be mentioned. First, we did not have data available about many important factors such as frailty and functional impairment [7, 23], socio-economical status [36], palliative care, cognitive impairment [37], use of medications or whether patients were admitted from or discharged to nursing homes, rehabilitation facilities or their own homes. The possibility for interpretation in a causal framework [38] is therefore limited, and the focus of the current study is limited to identification of predictors. Second, the physician who cared for the patient during the admission did register the discharge diagnosis. The discharge diagnoses in the official charts might not necessarily be the prioritized task in a busy environment, and there might be misclassifications, however earlier studies have shown that the validity of diagnoses in Danish medical departments is reasonable [39]. The selection of patients for admission and discharge is guided by the physician’s evaluation of need of acute hospital care, in-hospital capacity and expectations regarding the prognosis if discharged early. The findings in our study may not be generalizable to other countries where health care is organized differently.

### Implications

We have identified factors associated with mortality in the 30 days after early discharge from hospitals. These factors do partly overlap with the factors associated with readmission identified in an earlier study of the same cohort [8], and with factors associated with mortality after hospitalizations of all lengths identified in earlier nationwide studies [16]. However, we have not presented a predictive performance model with estimations of how accurately these factors are able to predict mortality. It may therefore be difficult to apply our results clinically [40]. The variables associated with mortality in our study should therefore be evaluated in future prediction models with estimations of absolute risk for individual patients and with validations of the model [40].

Such data, possibly combined with biomarkers [41] and other screening systems [37] could be used to select groups of patients where targeted interventions could be evaluated [41]. High risk patient groups could be offered timely follow up face to face at home or in an outpatient clinic [42], a longer hospital admission, or discharge to rehabilitation facilities with frequent follow-up. Telemonitoring with electronic communication between the patient and the team is also an opportunity that has been and will be evaluated. Several telemonitoring programs have been evaluated in diagnoses as heart failure, however the results in randomized controlled trials have been conflicting [43].

## Conclusions

Three percent of older acutely admitted medical patients discharged within 24 h from hospital had died within 30 days after discharge. A high comorbidity burden, male gender, unmarried status and several primary diagnoses were associated with death after early discharge. We suggest that professionals should pay attention to some of the factors that we have identified as prognostic factors of death after early discharge during the discharge risk assessments.

## Data Availability

Data are stored by Aarhus University Hospital, Denmark, on servers with security according to the rules given by Danish law. Access to the data to another project will require a new permission from The Danish Health Data Authority. The corresponding author can be contacted for further information.

## Abbreviations

aHR: Adjusted hazard ratios
CCI: Charlson comorbidity index
CI: Confidence interval
CRS: Civil registration system
DNPR: Danish national patient registry
ICD-10: International classification of diseases 10th Edition
IQR: Interquartile range
LOS: Length of stay
NA: Not available
TIA: Transient ischemic attack
