# Correction to: Mortality of older acutely admitted medical patients after early discharge from emergency departments: a nationwide cohort study

**DOI:** 10.1186/s12877-021-02420-6

**Published:** 2021-09-29

**Authors:** Martin Aasbrenn, Christian Fynbo Christiansen, Buket Öztürk Esen, Charlotte Suetta, Finn Erland Nielsen

**Affiliations:** 1 CopenAge – Copenhagen Center for Clinical Age Research, University of Copenhagen, Copenhagen, Denmark; 2 Geriatric Research Unit, Department of Geriatric and Palliative Medicine, Copenhagen University Hospital, Bispebjerg and Frederiksberg, Copenhagen, Denmark; 3 Department of Clinical Epidemiology, Aarhus University Hospital, Aarhus, Denmark; 4 Geriatric Research Unit, Department of Medicine, Herlev-Gentofte Hospitals, Herlev, Denmark; 5 Department of Emergency Medicine, Copenhagen University Hospital, Bispebjerg and Frederiksberg, Copenhagen, Denmark; 6 Department of Emergency Medicine, Slagelse Hospital, Slagelse, Denmark; 7 Copenhagen Center for Translational Research, Copenhagen University Hospital, Bispebjerg and Frederiksberg, Copenhagen, Denmark

**Correction to: BMC Geriatrics 21, 410 (2021)**

**https://doi.org/10.1186/s12877-021-02355-y**

Following publication of the original article [1], the authors reported some incorrect lines in Table 4.

The original article [1] has been updated.
